# Polypyrrole/carbon dot nanocomposite as an electrochemical biosensor for liquid biopsy analysis of tryptophan in the human serum of normal and breast cancer women

**DOI:** 10.1007/s00216-023-04784-7

**Published:** 2023-07-04

**Authors:** Fatma A. M. Abdel-aal, Rania M. Kamel, Asmaa A. Abdeltawab, Fardous A. Mohamed, Abdel-Maaboud I. Mohamed

**Affiliations:** 1 Pharmaceutical Analytical Chemistry Department, Faculty of Pharmacy, Assiut University, Assiut, 71526 Egypt; 2 Clinical Oncology and Nuclear Medicine Department, Faculty of Medicine, Assuit University, Assiut, 71526 Egypt

**Keywords:** Biomarkers, Carbon dots, Human serum, Polypyrrole, Square wave voltammetry, Tryptophan

## Abstract

Liquid biopsy analysis represents a suitable alternative analysis procedure in several cases where no tumor tissue is available or in poor patient conditions. Amino acids can play a crucial role in aiding cancer diagnosis. Monitoring of tryptophan (Trp) catabolism can aid in tracking cancer progression. Therefore, a novel nanocomposite was fabricated using overoxidized polypyrrole film doped with nano-carbon dots (nano-CDs) on the pencil graphite electrode (PGE) surface for sensitive evaluation of Trp in human serum. Using square wave voltammetry (SWV), the overoxidized polypyrrole/carbon dots/pencil graphite electrode (Ov-Ox PPy/CDs/PGE) achieved excellent electrochemical catalytic activity for evaluating Trp. The modified electrode, known as Ov-Ox PPy/CDs/PGE, demonstrated superior electrochemical catalytic activity compared to bare PGE, CDs/PGE, PPy/PGE, and PPy/CDs/PGE for evaluation of Trp. The method’s excellent sensitivity was confirmed by the low limits of detection (LOD = 0.003 μmol L^−1^) and limit of quantitation (LOQ = 0.009 μmol L^***−***1^). The biosensor that was developed can measure tryptophan (Trp) levels in the serum of both healthy individuals and female breast cancer patients with high accuracy and sensitivity. The results indicate that there is a significant difference, as shown by the *F*-test, between healthy individuals and those with breast cancer. This suggests that Trp amino acid could be an essential biomarker for cancer diagnosis. Consequently, liquid biopsy analysis presents a valuable opportunity for early disease detection, particularly for cancer.

**Graphical abstract:**

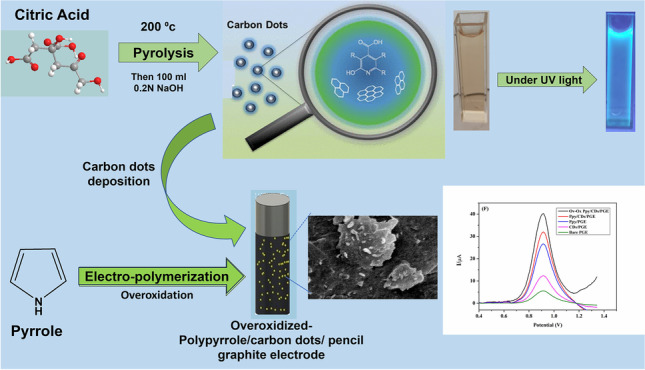

**lementary information:**

The online version contains supplementary material available at 10.1007/s00216-023-04784-7.

## Introduction

Tryptophan (Trp) is one of the essential amino acids for humans that must be supplied in diet [1]. Its catabolism is a well-studied process in cancer that plays a significant impact on immune system modulation. It can be metabolized by two pathways. The determining enzymes for the first pathway are tryptophan hydroxylase which leads to the synthesis of neurotransmitter serotonin and neurohormone melatonin [1, 2]. So Trp has a role in mood-related disorders such as depression [2, 3]. The other pathway is the kynurenine pathway that is the main pathway for its metabolism which produces kynurenines, which are immunomodulatory, neuroprotective, and neurotoxic intermediates.

The enzyme indoleamine 2,3–dioxygenase 1 (IDO1) initiates this metabolic pathway by converting Trp to formylkynurenine (NFK), which is then converted to kynurenine (Kyn, the first stable metabolite of the kynurenine pathway) [1]. Trp is very important for human health and any change in its plasma levels (around 31–83 µmol L^−1^) may be related to a number of disorders such as pellagra, chronic kidney diseases, Parkinson’s disease, and metabolic, mental, and sleep disorders [4, 5].

Breast glandular tissue contains cells called epithelium that line the ducts (85%) and lobules (15%), where breast cancer can start [6]. Breast cancer remains the most commonly diagnosed cancer in the world, with over 7.8 million women who have received a diagnosis within the past 5 years still alive [6]. The possibility of improving the prognosis of breast cancer by detecting it early has led to a growing need for global research on biomarkers at various stages of the disease’s development [7]. Among those biomarkers are the aromatic amino acids especially Trp [7, 8]. Several studies have demonstrated a correlation [9–15] between cancer development and Trp plasma or serum levels that suggest increased catabolism of Trp in cancer patients [9–14]. This leads to a decrease in Trp levels and an increased Kyn/Trp ratio, which may contribute to the immunosuppression of cancer patients by promoting the differentiation of T-regulatory cells with potential immunosuppressive effects.

There are several analytical methods for Trp quantitative determination such as spectrophotometric methods [15, 16], spectrofluorimetric [17–19], high-performance liquid chromatography (HPLC) [20–25], and electrophoretic [26] and electrochemical methods [27–33]. The utility of electrochemical techniques becomes highly trending due to their high accuracy, high sensitivity, and simplicity [33–35]. Recently, pencil graphite electrodes are widely used as they are cheap and available and have remarkable physical and chemical properties [36–38].

Carbon dots have emerged as a novel form of carbon-based nanomaterials and earned significant attention in recent years due to their exceptional qualities, including high stability, low-cost fabrication, eco-friendliness, excellent photoluminescence properties, high surface area, and high electrical conductivity. These dots also have numerous functional groups such as carboxyl, hydroxyl, and amino groups, which could enhance redox response and make them a promising source for many research studies [39–41].

Polypyrrole (PPy) is a compelling polymer due to its biocompatibility, high stability, sensitivity, malleability, and high electrical conductivity, making it an attractive prospect for various applications. Furthermore, it can operate under neutral pH conditions, as reported in studies before [42–46]. The inclusion of such a conductive polymer raises the proposed method’s conductivity and sensitivity levels. Overoxidation of polypyrrole (Ov-Ox PPy) enriches the resulting film with electron-rich oxygen-based groups, such as carbonyl and carboxyl groups, which enhance the accumulative effect of the Ov-Ox PPy film. The addition of high conductivity and accumulative effects of Ov-OX PPy film on the large surface area derived from synthesized carbon dots nanotubes results in a highly applicable and sensitive method for determining Trp and using it as a biological marker in cancer patients.

Therefore, we aim in this work to develop a novel electrochemical sensor for the biological determination of Trp in serum samples of both healthy and breast cancer–suffering females. We fabricate a new pencil graphite electrode modified with overoxidized polypyrrole/deposited carbon dot film (Ov-Ox PPy/CDs film). This film provides a porous coating surface with molecular sieve properties. This film increases the selectivity and sensitivity of the method for the determination of Trp in serum samples. The method is proposed for the determination of Trp as an essential biomarker in healthy and breast cancer women and to determine if there is a significant difference between Trp serum levels in both cases.

## Experimental

### Chemicals and materials

All chemicals used in this work were of analytical grade and were used without further purification. Trp, pyrrole, phosphoric acid, boric acid, and sodium hydroxide were purchased from Sigma-Aldrich (Steinheim, Germany). Potassium ferricyanide and sulfuric acid were obtained from Fluka, Buchs, Switzerland. Hydrochloric acid, glacial acetic acid, and citric acid were purchased from El-Nasr, Egypt. The supporting electrolyte used in the electrochemical detection of Trp was 0.04 M Britton Robinson buffer (B.R.) and the pH was adjusted by 0.2 M NaOH. All solutions were prepared using double distilled water.

### Instrumentation

The electrochemical work was done using a Princeton VersaSTAT MC (VersaSTAT 3, Model RE-1, Princeton Applied Research, AMETEK, USA). A three-electrode system was used that consists of a bare/modified PGE, Ag/AgCl (saturated KCl), and a platinum wire as the working, reference, and auxiliary electrodes, respectively. The surface morphologies of the bare and modified electrodes were studied by scanning electron microscopy (SEM; JEOL JSM-7500F instrument; Oxford, USA). The prepared carbon dots were investigated using transmission electron microscopy (TEM) (TECNAI G^2^spirit TWIN microscope, operating at 120 kV and conducted by VELETA camera). Specimens for TEM are prepared by ultrasonic dispersion of the carbon dot solution and putting a droplet of the solution on a copper microscope grid covered with carbon.

A Nicolet 6700 FT-IR advanced gold spectrometer, supported with OMNIC 8 software (Thermo Electron Scientific Instruments Corp., WI, USA), was used for data processing. The spectrophotometric measurements were carried out using a double‐beam UV-VIS spectrophotometer (Shimadzu, Kyoto, Japan) model UV‐1601 PC connected to an IBM‐compatible computer, with UVPC personal spectroscopy software version 3.7. The spectrofluorimetric spectrum was measured using a Shimadzu RF-5301 spectrofluorometer **(**Kyoto, Japan). A pH meter (Hanna Instruments, Sao Paulo, Brazil) was utilized for recording the pH values of the buffer solutions. All the measurements were conducted at room temperature.

### Experimental procedures

#### Synthesis of carbon dots (CDs)

The method of carbon dot synthesis is based on the thermal decomposition procedures previously reported in the literature [47]. Briefly, 2.0 g of citric acid (as the carbon source) was pyrolyzed on the hot plate at 200 °C for 30 min and then neutralized with a solution of 0.2 N NaOH. The synthesized carbon dots were examined using a transmission electron microscope (TEM) to determine their shape and particle size.

#### Preparation of the different modified electrodes

Pencil graphite electrode bare (PGE bare) was first immersed overnight in the prepared solution of carbon dots. The modified CDs/PGE electrode was dipped into a 10-mL cell containing 0.05 M pyrrole solution (dissolved in 0.1 M KClO_4_) and then cyclic voltammetry was carried out in a potential range from  − 0.9 to 1.0 V at a scan rate of 0.1 V/s for 10 cycles.

Subsequently, after electropolymerization of pyrrole, the modified polypyrrole/carbon dots/PGE (PPy/CDs/PGE) electrode was washed carefully with distilled water; then, the overoxidation process for the prepared polypyrrole film was performed by cyclic voltammetry in 0.05 N NaOH solution in the potential range  − 0.9 to 1.0 V with a scan rate at 0.1 V/s for succussive 10 cycles. The modified overoxidized PPy/CDs/PGE was washed thoroughly with distilled water; then, it became ready for measuring Trp in Britton Robinson buffer at pH 2.7.

#### Electrochemical procedures

The modified electrode (Ov-Ox PPy/CDs/PGE) was used in square wave voltammetry to measure Trp at a pH of 2.7 in B.R. buffer, with an accumulation potential of 0.2 V, frequency of 250 Hz, step height of 9 mV, pulse height of 10 mV, and deposition time of 90 s. In this study, the reference electrode used for measuring all potentials was an Ag/AgCl electrode.

#### Electrochemical impedance spectroscopy (EIS)

The electron transfer at the electrode-solution boundary was investigated using electrochemical impedance spectroscopy (EIS) study. EIS for the prepared electrodes was studied in 1.0 mmol L^−1^ of K_3_[Fe(CN)_6_] solution in 0.5 mol L^−1^ KCl, which was adjusted by 1.0 mol L^−1^ HCl to pH 2.5 at the open circuit potential over the range 1.0 Hz to 100 kHz. The Nyquist plots to compare the unmodified and modified electrodes are used to illustrate the EIS results. The diameter of the semi-circular portion used to study the electron transfer limited process was equal to the charge transfer resistance (Rct).

#### Sample collection and preparation

Samples were obtained from fourteen healthy control women (20–55 years) and 17 breast cancer women patients (aged 30–60 years) from the Assiut University Hospital. The healthy control volunteers had at least 2 weeks before the trial without experiencing any physical illness. The study protocol was approved by the Medical Ethics Committee of Assiut University (*IRB approval number is 17300799*). Human blood serum samples were collected from both healthy female volunteers and breast cancer females by collecting the venous blood following overnight fasting. The tubes were allowed to stand for about 30 min followed by centrifugation at 3000 rpm for 15 min. 0.5 mL of the obtained separated serum samples was first diluted 20 times with BRP (0.04 M, pH 2.75) then moved to the electrochemical cell for the analysis. After that, the electrochemical procedure was performed as discussed before in “Electrochemical procedures.”

## Results and discussion

### Characterization of the synthesized carbon dots

**Fig. 1:**
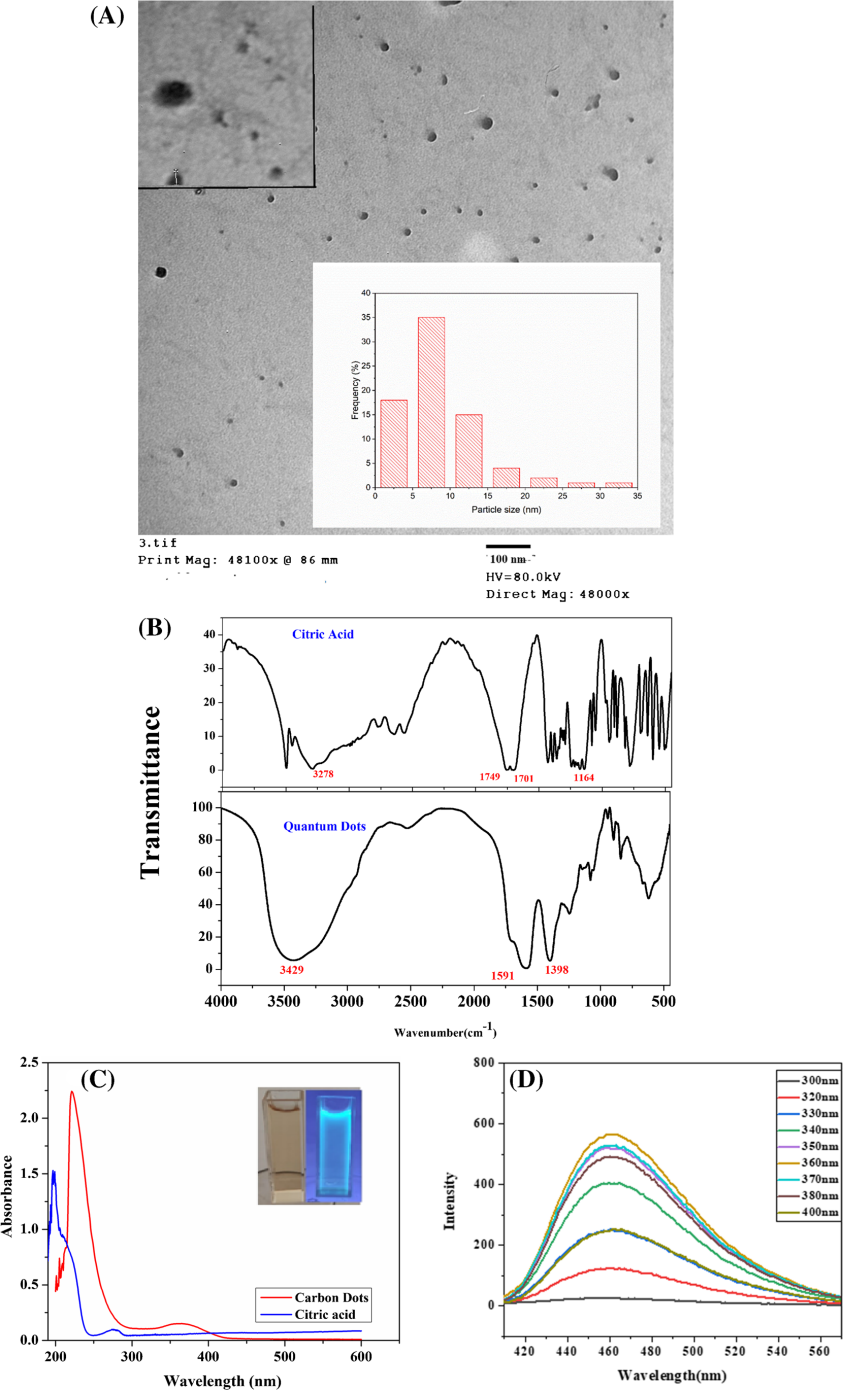
**A** Typical TEM picture of CDs prepared using 2.0 g of citric acid (CA). *Inset*: high magnification TEM picture demonstrated the morphology of the nanoparticles prepared and carbon dot size distribution determined with ImageJ software. **B** Typical FT-IR spectra of citric acid (CA) and the synthetic carbon dots (CDs). **C** UV spectrum of the synthetic carbon dots and citric acid. *Inset:* photographs under white light (brown color) and UV lamp 365 nm (blue color) and **D** emission spectra of the synthesized carbon dots with different excitation wavelengths (300–400 nm)

Because of its great resolution, a transmission electron microscope (TEM) can be used to identify the ultrastructure of samples. This technique can be used to investigate the morphology of the prepared carbon dots in order to learn more about their shape, size, and dispersion. In the captured images, the synthesized carbon dots appeared as a round or spherical shape with a particle size from  ~ 10 to  ~ 50 nm as shown in Fig. 1A. To verify the particle size and their distribution, ImageJ (a popular image analysis software) was used to measure the particle size of carbon dots (CDs) in transmission electron microscopy (TEM) images. The software allows users to analyze digital images and quantify various properties, including particle size, shape, and distribution.

To verify the surface functional groups of the manufactured C-dots, FT-IR spectra were used. According to Fig. 1B, there are clear changes between the FT-IR spectra of citric acid and the synthesized CDs. A broadening of O-H stretching at 3292 cm^−1^, C = O stretching at 1749 and 1701 cm^−1^, and C-OH stretching at 1164 cm^−1^ was detected in citric acid (precursor). While the synthetic carbon dots exhibit peaks at 1591 and 1398 cm^−1^ that are thought to be caused by the stretching vibrations of the carboxylate group in symmetric and asymmetric patterns, respectively [48, 49]. Furthermore, the broad intense peak at 3429 cm^−1^ can be ascribed to the O-H stretching vibration of the carbon dots [50, 51].

The optical properties of the synthesized carbon dots with citric acid as a starting material were investigated and explained using optical spectra. The absorption maxima of the CDs can be observed in the UV-visible absorption spectra (Fig. 1C) at 238 nm and 365 nm, respectively. The first peak at 238 nm can be attributed to π–π* transitions while the broad peak at 365 nm can be attributed to the n–π* transitions of the carbonyl bond (C = O) [52, 53]. The spectrum of citric acid shows a complete conversion of the citric acid to CDs. This suggests that the synthesis process was successful in producing CDs from citric acid. The manufactured CDs also have strong fluorescent characteristics. The maximum excitation wavelength for the manufactured CDs is 360 nm, and the maximum emission wavelength is 460 nm. The excitation-dependent emission phenomena were demonstrated when excited between 300 and 400 nm as shown in Fig. 1D. It’s possible that this is related to how the different sizes and surface states are distributed. The fluorescence intensity changed with changes in excitation wavelength reaching a maximum intensity at *λ*_ex_ = 360 nm after that the fluorescence intensity decreased. The change in excitation wavelength did not affect the position of emission wavelength at 460 nm. This may be attributed to the monodisperse nature of the particles synthesized [54].

### SEM characterization of the modified electrodes

**Fig. 2:**
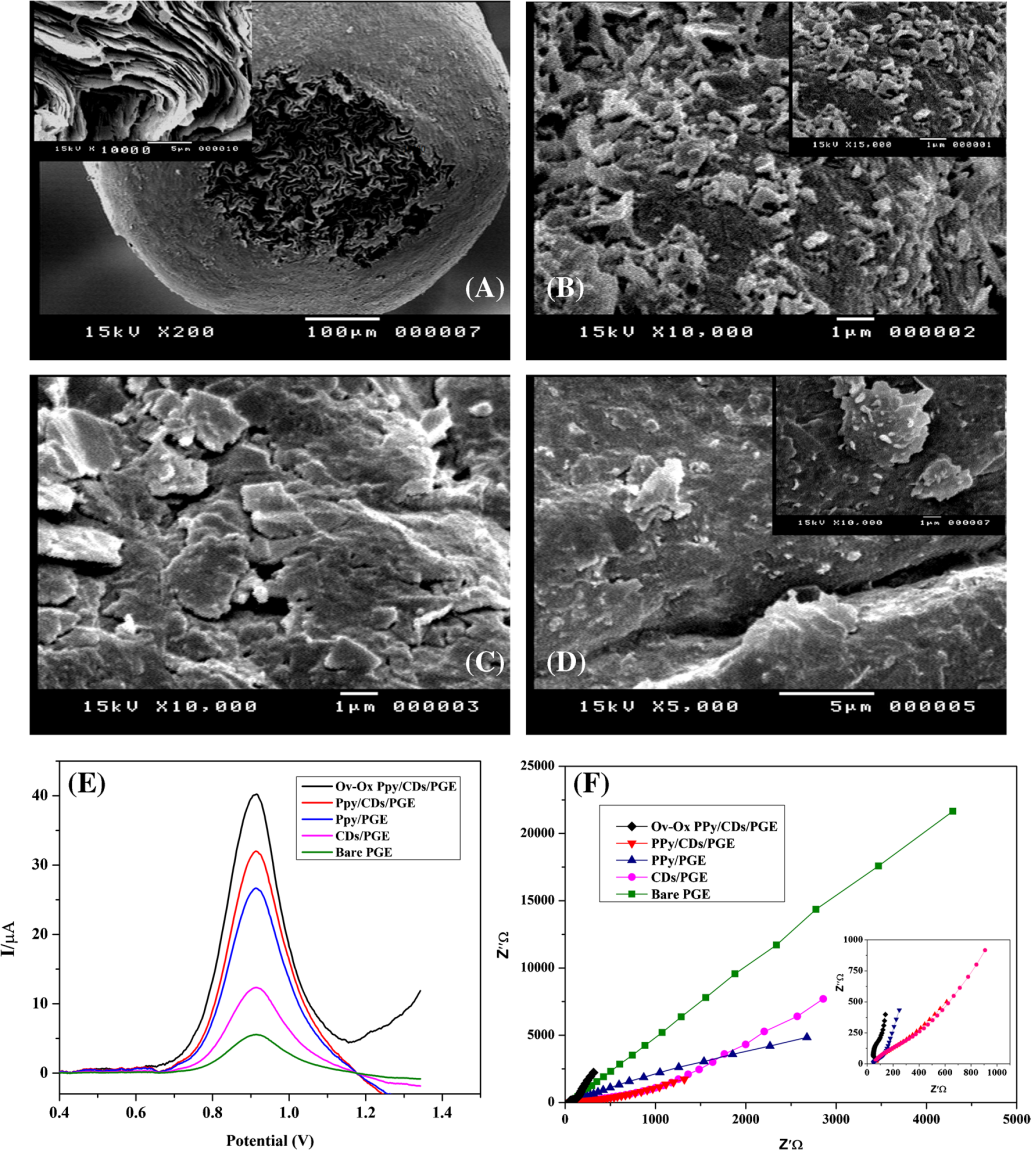
SEM pictures of **A** bare PGE. *Inset:* high magnification SEM picture indicating the grooves and the craves at the surface. **B** Carbon dot–modified PGE (CDs/PGE). *Inset:* high magnification SEM picture indicating the small aggregations at the electrode surface. **C** Polypyrrole-modified PGE (PPy/PGE), and **D** PGE modified with carbon dots and overoxidized polypyrrole polymer (Ov-Ox PPy/CDs/PGE). *Inset:* high magnification SEM picture indicating the scales scattered within the CD aggregations. **E** Square wave voltammograms of 4.0 × 10^−5^ mol L^−1^ Trp solution at bare PGE, CDs/PGE, PPy/PGE, PPy/CDs/PGE, and Ov-Ox PPy/CDs/PGE. **F** Nyquist plot that indicates the impedance of the bare and different modified electrodes. *Inset:* high magnification of the first part of the EIS spectra

By comparing the different electrodes (bare PGE, CDs/PGE, PPy/PGE, and Ov-Ox PPy/CDs/PGE) under the electron microscope, it was found that bare PGE has large pores, grooves, and cavities and staked flakes at different sizes as shown in Fig. 2A. As compared to bare PGE, the CDs/PGE electrode indicated that the deposited CDs appeared as small aggregations on the surface of PGE (Fig. 2B), enhancing the electrode’s surface area. While the surface of PPy/PGE developed enormous flakes or scales that covered the whole surface of the electrode after pyrrole polymerization on the surface of PGE. This could have a significant impact on the surface area and conductivity of the electrode (Fig. 2C). The Ov Ox-PPy/CDs/PGE combined the advantages of the two electrodes. The electrode surface is covered with polypyrrole scales with scattered tiny, protruding carbon dot particles inside of it, as shown in Fig. 2D. The electrode’s exposed surface area increased noticeably as a result.

### Electrochemical behavior of the bare and different modified electrodes

SWV diagrams for 4.0 × 10^−5^ mol L^−1^ Trp in 0.04 M BRP (pH 5.0) on both the unmodified and different modified electrodes are shown in Fig. 2E. It can be demonstrated that on utilizing the bare electrode, a very small and broad peak attributed to the electrooxidation of Trp appeared. While, on utilizing the other electrodes (CDs/PGE, PPy/PGE, and PPy/CDs/PGE without overoxidizing polypyrrole), the peak current of Trp gradually increased. While the overoxidized PPy/CDs/PGE exhibits a synergistic impact of both modifiers, including the benefits of polypyrrole overoxidation and the addition of more functional groups to the electrode’s surface, which improves peak shape and intensity.

Electrochemical impedance spectroscopy (EIS) is a powerful analytical technique used to study the electrical properties of electrochemical systems, including bare and modified electrodes. By applying an alternating current (AC) signal to the electrode and measuring the resulting potential response, EIS can provide information about the resistance and capacitance of the electrode-electrolyte interface, as well as the kinetics of charge transfer reactions. When comparing the behavior of bare electrodes and modified electrodes using EIS, several parameters can be examined. For example, the impedance or resistance of the electrode-electrolyte interface can be measured, which can provide insights into the effectiveness of surface modifications in increasing or decreasing the electrode’s reactivity. The charge transfer resistance is inversely proportional to the rate of electron transfer at the electrode surface. In other words, a higher charge transfer resistance indicates a slower rate of electron transfer, while a lower charge transfer resistance indicates a faster rate of electron transfer.

The different behaviors of the bare electrodes and different modified electrodes were compared also using the electrochemical impedance spectroscopy (EIS). Figure 2F shows Nyquist plots for PGE bare, CDs/PGE, PPy/PGE, PPy/CDs/PGE, and Ov-Ox PPy/CDs/PGE. The semi-circular model was more noticeable in the Ov-Ox PPy/CDs/PGE other than the other electrodes, whereas the linear model was more prominent in the CDs/PGE, PPy/PGE, and PPy/CDs/PGE electrode especially at higher frequencies values. Ov-Ox PPy/CDs/PGE was observed to have the lowest value after the circle fitting of the curves to determine the values of charge transfer resistance [55, 56]. For PGE bare, CDs/PGE, PPy/PGE, PPy/CDs/PGE, and Ov-Ox PPy/CDs/PGE, the fitted values of Rct were found to be 8241, 4734, 2617,1267and 616 ohm. The results indicated that the polypyrrole polymer and carbon dots adsorbed on the PGE surface enhance electron transfer and reduce resistance at the electrode-solution interface. Therefore, the use of surface modifications using carbon dots and polypyrrole polymer film can be an effective strategy for improving the performance of electrochemical systems, particularly in applications where efficient electron transfer is critical.

### Electrochemical characterization of the bare and different modified electrodes

The electrochemical activities of the unmodified and modified electrodes were examined in a solution of 1.0 mmol L^−1^ K_3_Fe(CN)_6_ in 0.5 mol L^−1^ KCl. Figure 1S (A–D) shows the CV of the various electrodes at different scan rates. When compared to the other electrodes, it could be demonstrated that the redox peaks at the Ov-Ox PPy/CDs/PGE electrode increased significantly. It was also observed that the peak current of K_3_Fe(CN)_6_ increased linearly with scan rates squared. Randles–Sevcik equation could be used for the reversible process as follows:

$${I}_{\mathrm{p}}=2.69\times {10}^{5} {\mathrm{An}}^{3/2}{\mathrm{Dr}}^{3/2}{C_0\upsilon }^{1/2}$$

where *C*_0_ is the reactant concentration (1 mM K_3_Fe(CN)_6_), *I*_p_ is the peak current, *A* is the surface area of the electrode, *n* is the number of electrons included (*n* = 1) in the K_3_Fe (CN)_6_ redox system), DR is the K_3_Fe(CN)_6_ diffusion coefficient (7.6 × 10^−6^ cm2 s^−1^), and *υ*^1/2^ is the square root of the scan rate.

The surface area *A* for each electrode can be determined using the Randles–Sevcik equation using the slope obtained from the plot of *I*_p_ versus *υ*^1/2^ at the various scan rates for the four prepared electrodes; the surface area *A* for each electrode can be calculated using the Randles–Sevcik equation. It was clear that *A* values were 8.78, 12.78, 18.19, and 20.96 mm^2^ for bare PGE, CDs/PGE, PPy/PGE, and Ov-Ox PPy/CDs/PGE respectively.

According to the results, when compared to the other produced electrodes, Ov-Ox PPy/CDs/PGE had the highest surface area. This increase in peak current could be correlated with the improvement in effective surface area.

### Optimization of the experimental conditions

#### Carbon dot deposition on the surface of the bare electrode

To establish the optimal time for the deposition of a carbon dot layer on the electrode surface, PGE was dipped in the CD solution for various periods of time, including 10, 15, 30, 60, and overnight. It was found that overnight immersion of the PGE produced the highest and most repeatable current intensity for the peak of Trp oxidation. As stated in “Electrochemical behavior of the bare and different modified electrodes,” CDs’ high surface area and high surface-to-volume ratio provide a high degree of contact with Trp molecules. This behavior is attributed to electrostatic interactions and hydrogen bonding that occurred at the electrode surface. The deposited CDs can be rich in functional groups such as carbonyl (C = O) and hydroxyl (-OH) groups that increase the surface area and encourage the electrostatic interactions at the surface of the modified electrode (CDs/PGE) [57].

#### Factors affecting the electropolymerization of pyrrole

The amount and performance of the polymer films produced as a result of this sophisticated electrochemical process used to prepare these conducting polymers are influenced by a number of variables. The kind and amount of the monomer, the cell environment, the solvent, the applied voltage, and the pH are among the variables that significantly affect the electrooxidation reaction and the film’s quality [58]. Therefore, optimizing these parameters in a single experiment is difficult.

Several polymerization conditions of pyrrole were studied to indicate its influence on Trp oxidation such as polymerization medium, concentration of pyrrole, deposition potential, and the number of cycles as shown in Fig. 2S (A, B, and C). Trp concentration used in all experiments was 4.0 × 10^−5^ mol L^−1^ at pH 3.0 of 0.04 mol L^−1^ B.R. buffer.

Pyrrole polymerization was tested in water and buffer solutions with a pH range from 2.7 to 7.0 that contained 0.04 mol L^−1^ B.R. buffer. It was found that the best results were obtained using water as the medium for polymerization.

Different pyrrole concentrations were investigated also to obtain the optimum concentration that gives the best results. As seen in Fig. 2SA, a range of concentrations from 0.01 to 0.08 mol L^−1^ were tried to be polymerized on the electrode’s surface. The oxidation peak current of Trp was shown to increase with pyrrole concentration, reaching a maximum at 0.05 mol L^−1^ of pyrrole. Above this concentration, the current intensity was reduced because the electrode surface was blocked.

The effect of polypyrrole deposition potential was also investigated using different potentials ranging from  − 1.0 to 0.0 V to reach the best value suitable for its deposition. Taking the Trp oxidation peak as a guide, it was found that the best Trp oxidation current and peak shape were obtained at  − 0.9 V, which was selected as the optimal potential to be used for the remainder of the investigation as shown in Fig. 2SB.

The number of polymerization cycles has a significant impact on the thickness of the polypyrrole layer that is deposited on the electrode surface. As shown in Fig. 2SC, the Trp oxidation current response increased as the number of cycles increased, reaching a maximum value at 10 cycles. The oxidation sites on the electrode surface are blocked when the number of cycles increases over 10, which results in a reduction in the current responsiveness.

#### Polymerization and overoxidation mechanism

**Fig. 3:**
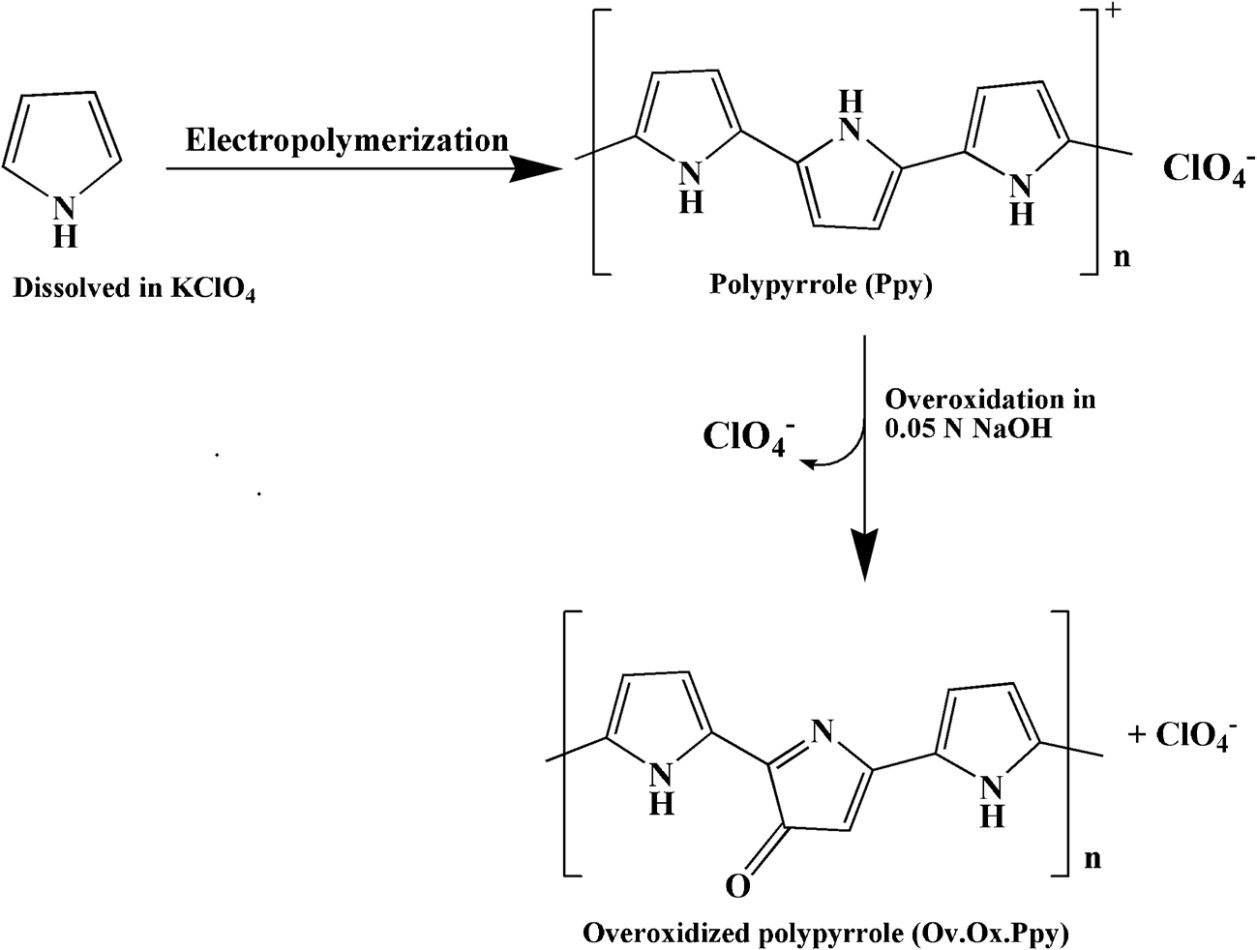
Electropolymerization of pyrrole and overoxidation of the polymer on the surface of the electrode

Unique characteristics of conducting polypyrrole film (PPy) are its simplicity in synthesis (one-step procedure), environmental stability, and high electrical conductivity [59]. The monomer units of pyrrole are primarily bonded at α-α positions to form the polymer as shown in Fig. 3. Because of the alternating single and double bonds, which generate some delocalization of the electron density within the molecule, PPy is conductive [60]. The main benefit and advantage of the electrochemical polymerization of pyrrole on the surface of the electrode is the ability to control the thickness of the film deposited. This can be done by optimization of the possible affecting factors during its synthesis such as monomer concentration, pH of the medium, deposition potential, and number of cycles used for polymerization. All these factors are optimized to reach to the best conditions used for polymerization that give the highest oxidation peak current for Trp. After deposition of PPy polymer film, overoxidation is applied. Overoxidation is one of the irreversible reactions that are applied on PPy molecule. High-density carbonyl groups (C = O) generated at the electrode surface as a result of anodic overoxidation that carried out in an alkaline media, as shown in Fig. 3. This behavior (ion exchange equilibrium mechanism) promoted the accumulation of cationic species on the electrode’s surface (like Trp in an acidic medium). The preferential interaction between the investigated molecule and the overoxidized polymer is caused by the presence of these carbonyl groups (Trp).

#### Scan rate effect on the electrooxidation of Trp using the modified Ov-Ox PPy/CDs/PGE

**Fig. 4:**
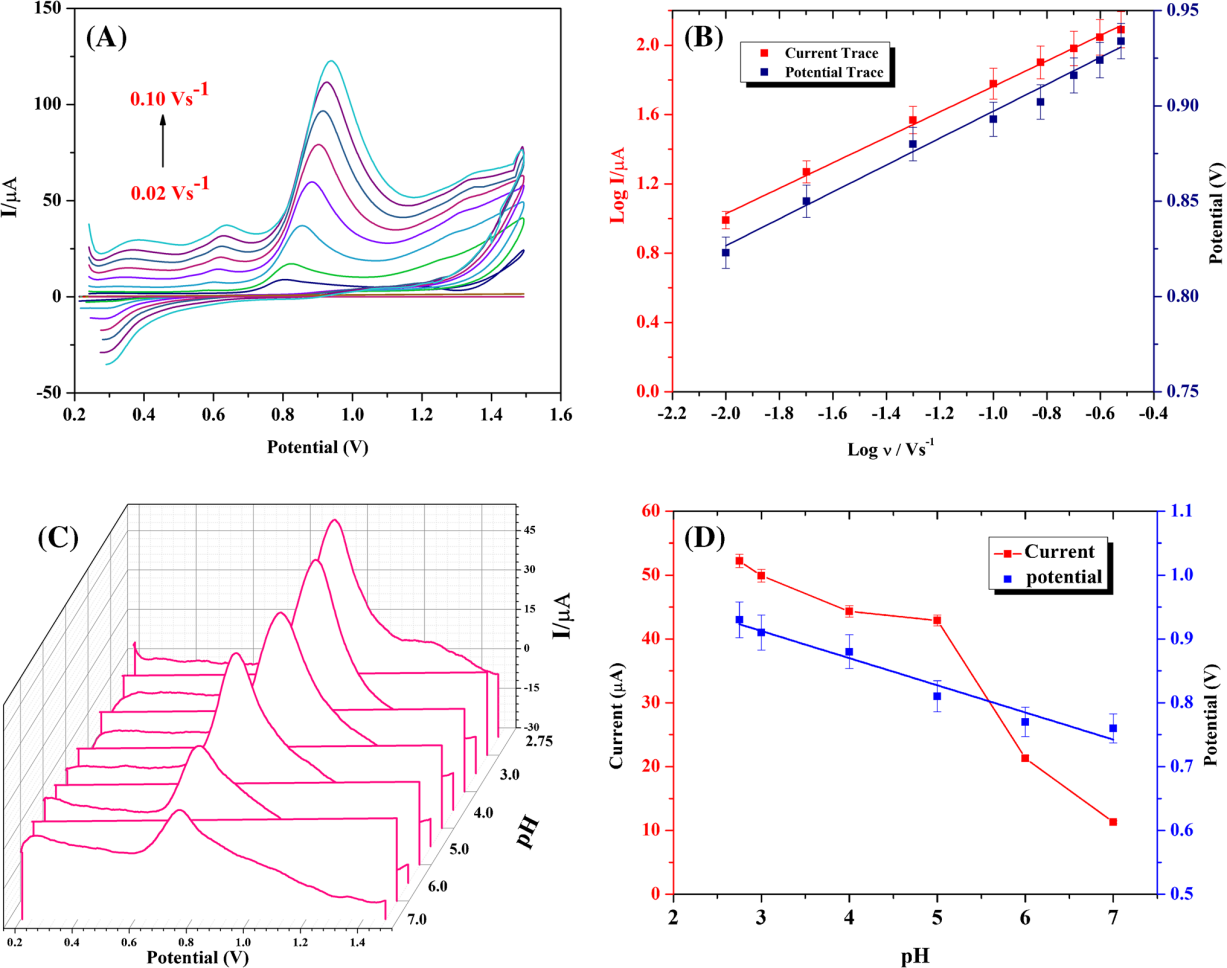
**A** Cyclic voltammograms to investigate the effect of different scan rates (0.02 to 0.10 V s^−1^) on the electrochemical response of 1.0 × 10^−4^ mol L^−1^ Trp solution. **B** Correlation between log scan rate (log *ν*) and log peak current (log *I*) or potential (V). **C** 3D square wave voltammograms for the influence of different pHs on the oxidation peak of 4.0 × 10^−5^ mol L^−1^ Trp solution ranged from 7.0 to 2.75. **D** Effects of pH on the peak potential and current of 4.0 × 10^−5^ mol L^−1^ Trp solution at PPy/CDs/PGE. Deposition time, 90 s; step height, 9 mV; pulse height, 10 mV; deposition potential, 0.2 V; and frequency, 250 Hz

Generally, the electrooxidation of Trp exhibits only one anodic peak. Although one small reduction (Ep = 0.28 V) and two weak oxidation peaks (Ep = 0.35 and 0.6 V) were observed as shown in Fig. 4A. In this study, we have focused on the main peak (Ep = 0.9 V) that corresponds to the oxidation of Trp and increased with increasing its concentration. While the other small peaks are explained before by Sakthivel et al. [61] and Oikawa and Yonemitsu [62]. They reported that these peaks are attributed to the selective oxidation of the side chain at C-3 indole. The influence of the scan rate on the oxidation of Trp on the surface of Ov-Ox PPy/CDs/PGE was investigated by CV. Utilizing the Ov-Ox PPy/CDs/PGE, several scan rates between 0.02 and 0.10 Vs^−1^ were used to determine how they affected the oxidation peak current of Trp (1.0 × 10^−4^ mol L^−1^) in B.R. buffer (pH = 2.75) as shown in Fig. 4A. It is critical to establish whether adsorption or diffusion is controlling the reaction on the surface of the modified electrode. The following equation shows a linear relationship between the oxidation peak current (*I*_p_) μA and the square root of the scan rate (*ν*^1/2^) (Vs^−1^)^1/2^:

$$(\mathrm{Ip}_{\mathrm{Trp}})(\mu \mathrm{A}) = 365.79 \;\mathrm{v} ^{1/2} (\mathrm{Vs}^{-1})^{1/2} -33.66 \qquad (\mathrm{r} = 0.9930)$$

To support the mechanism on the surface of the modified electrode, the correlation between the logarithms of the scan rate and the oxidation *I*_p_ (log *ν*, log *I*_p_) was investigated. The adsorption diffusion-controlled mechanism is confirmed by the linear relationship between them (Fig. 4B), with a slope of 0.888, which is shown in the following equation:

$$(\log \,\mathrm{IPT}_{\mathrm{rp}})(\mu \mathrm{A}) = 0.89 \log \mathrm{v} \; (\mathrm{Vs}^{-1}) + 2.82 \qquad (\mathrm{r} = 0.9888)$$

Additionally, it was found that Ep and log *ν* have a linear relationship, and that as applied scan rates are increased, peak potential values change to greater values (Fig. 4B).

This change demonstrated that Trp oxidation on the changed electrode surface is irreversible.

A range of scan rates (0.02 to 0.10 Vs^−1^) were used. The following equation shows the linear relationship:

$$\mathrm{Ep}(\mathrm{V}) = 0.08 \log \mathrm{v} (\mathrm{Vs}^{-1}) + 0.96\; (r = 0.9968)$$

According to the Lavarion theory [63], the following equation can be used to determine how many electrons are involved in the Trp oxidation process for the entirely irreversible electrode process:

$$\mathrm{Slope}=2.303\mathrm{RT}/\mathrm{\alpha nF}$$

where *n* is the number of electrons participating in the rate-determining step during the oxidation, *R* is the universal gas constant (8.314 J mol^−1^ K^−1^), *T* is the absolute temperature (298 K), *α* is the transfer coefficient, and *F* is the Faraday constant (96,480 C mol^−1^). The number of electrons (*n*) was calculated using the slope of the correlation between Ep and log *ν*. The slope’s value is 0.077, and the correlated αn value is 0.76. At *α* = 0.5, it was assumed that *n* would be 1.52, which may be rounded to two electrons and agrees with earlier reports [61, 64, 65].

#### Effect of pH and supporting electrolytes

The electrochemical behavior of the modified electrode can be influenced by a number of factors, including the pH of the solution. Changes in pH can affect the redox properties of the analyte or the modified electrode surface, which can in turn influence the electrochemical signals obtained. In Fig. 3D, it appears that the oxidation peak current of Trp on the modified electrode increases with decreasing pH, from pH 7.0 to pH 2.75. This suggests that the electrochemical response of the modified electrode is sensitive to changes in pH. To further understand the effect of pH on the electrochemical signals obtained from the modified electrode, it would be important to consider the redox properties of Trp and the modified electrode surface under different pH conditions. For example, changes in pH could affect the protonation state of the Trp molecule, which could in turn influence its electrochemical behavior. Alternatively, changes in pH could affect the charge density or surface chemistry of the modified electrode, which could influence the electrostatic interactions between the electrode surface and the analyte.

As illustrated in Fig. 4C and D, it was observed that the oxidation peak current of 4.0 × 10^−5^ mol L^−1^ Trp solution is high in the acidic medium with the maximum response at pH 2.75. Upon making a correlation between oxidation peak potential Ep and pH, it was found that shifting the solution pH from 2.75 to 7.0 caused the peak potential of Trp oxidation to shift to the negative portion according to the following equation:

$$\begin{array}{cc}\mathrm{Ep}\left(\mathrm{V}\right)=1.04-0.43\; \mathrm{pH}& \left(\mathrm{n}=6, \mathrm{r}=0.9813\right)\end{array}$$

As can be seen, the slope’s absolute value (0.043) was approximately close to the predicted value (0.059 V/pH at 298 K), suggesting that there were an equal number of protons and electrons involved in the electrochemical reaction. Different other acidic media were investigated such as 0.1, 0.01 M HCl and 0.1, 0.01 M H_2_SO_4_, and the best response of the oxidation current of Trp was obtained using the B.R. buffer (pH 2.75), so it was chosen for the subsequent experiments in this study.

The influence of anions in various supporting electrolytes on the oxidation current of Trp was also investigated. Different salts were examined such as KNO_3_, KCl, and KCLO_4_ in an equal concentration of 0.1 M, and the Trp oxidation current was monitored. It was found that the best current response was observed in the supporting electrolyte solution of 0.04 M B.R. buffer (pH 2.75) containing KNO_3_. Different concentrations of KNO_3_ (from 0.01 to 0.1 M) were studied to optimize the optimum concentration required to obtain the best result. It was found that a concentration of 0.05 is the optimum one with the highest peak current for Trp.

#### Effect of voltammetric parameters

##### Effect of accumulation potential and time

The accumulating potential of Trp was investigated in the range of  − 0.5 to + 0.4 V, as shown in Fig. 3SA. Trp’s accumulation potential was increased, causing an increase in peak current that reached a maximum at  + 0.2 V and subsequently decreased. This is why  + 0.2 V was chosen for further investigation. For accumulation time, as it is indicated in Fig. 3SB, when increasing the accumulation time, there was an increase in peak current response reaching the maximum at 90 s. Then, a decrease in the current response was monitored that may indicate the saturation of the electrode surface.

##### Effect of frequency, pulse height, and step height

Additionally, the 5–30 mV pulse height, 3–20 mV step height, and 10–250 Hz frequency of SWV variables were examined. It was discovered that the ideal combination for achieving the highest sensitivity and best peak shape was a pulse height of 10 mV, step height of 9 mV, and frequency of 250 Hz. Optimizing these parameters is important for achieving accurate and precise measurements in SWV-based analytical methods. The pulse height determines the amplitude of the voltammetric signal, while the step height determines the resolution of the measurement. The frequency of the SWV signal affects the sensitivity and noise level of the measurement.

### Oxidation mechanism

**Fig. 5:**
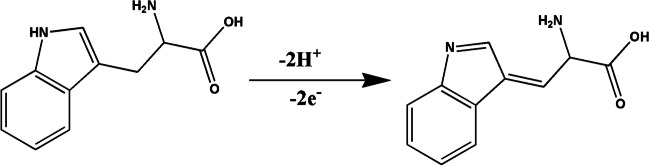
The reported mechanism of tryptophan oxidation

According to the previous literature [66, 67], Trp on the electrode surface undergoes an irreversible oxidation pathway. The achieved value of αn during the scan rate study demonstrated that two electrons were involved in the oxidation of Trp on the modified electrode, as previously mentioned in “Scan rate effect on the electrooxidation of Trp using the modified Ov-Ox PPy/CDs/PGE.” A slope of 42 mV per pH unit, which is near the theoretical value of 59 mV per pH unit. This slope indicates that the oxidation pathway of tryptophan on the modified electrode involves an equal number of protons and electrons, as shown in Fig. 5. Understanding the electrochemical behavior of tryptophan on the modified electrode is important for optimizing the electrode for use in analytical applications, such as the determination of tryptophan in biological samples.

### Analytical performance

**Fig. 6:**
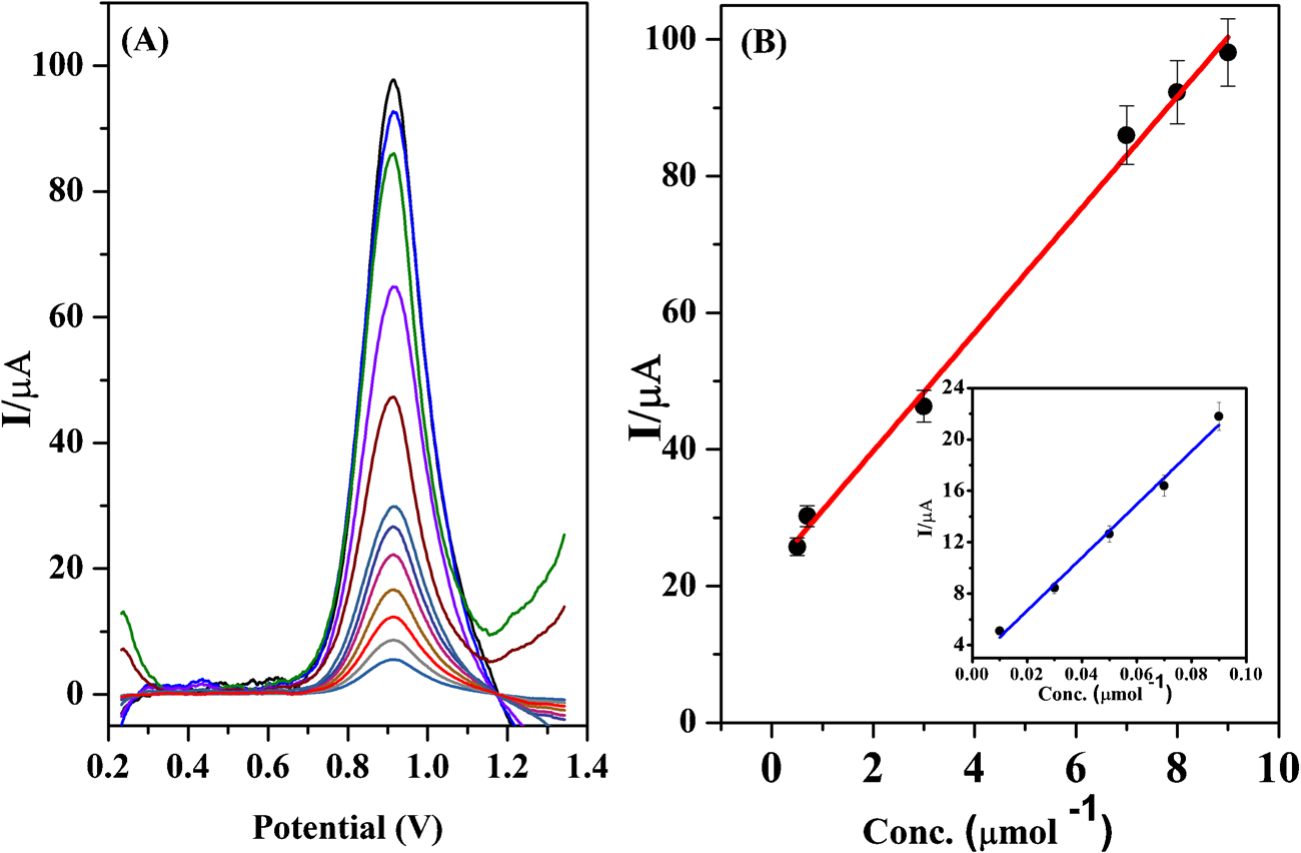
**A** Representative square wave voltammograms and **B** the corresponding calibration curves at PPy/CDs/PGE in B.R. buffer at pH 2.75 for two linear ranges (0.01 to 0.09 µmol L^−1^ and 0.5 to 9.0 µmol L^−1^) Deposition potential, 0.2 V; deposition time, 90 s; step height, 9 mV; frequency, 250 Hz; and pulse height, 10 mV

SWV was used to carry out the quantitative analysis of Trp. Under the optimal conditions, the Trp concentration was proportional to the oxidation peak current of Trp within two concentration ranges of 0.01 to 0.09 µmol L^−1^ and 0.5 to 9.0 µmol L^−1^, respectively, and with a linear strong correlation (*r* = 0.9996 and 0.9979 for the two calibration ranges). Table 1 and Fig. 6A and B show these results, and the regression equations are shown as follows:

**Table 1:** Regression equations parameters for quantitative determination of Trp in their standard solutions (*n* = 6)

Parameter	Value
Linearity range (µmol L^−1^)	0.01–0.09 0.50–9.00
LOD (µmol L^−1^)	0.003
LOQ (µmol L^−1^)	0.009
Intercept, *a* (µA) ± SD	2.97 ± 0.17 22.43 ± 1.64
Slope, *b* (µA, µmol^−1^ L) ± SD	190.31 ± 3.01 8.66 ± 0.28
Correlation coefficient (*r*)	0.9996 0.9979
Determination coefficient (*r*^2^)	0.9993 0.9958
Intra-day precision, *n* = 6 (7.0 µmol^−1^ L)	2.09
Inter-day precision, *n* = 18 (7.0 µmol^−1^ L)	3.33

$$\begin{array}{cc}{I}_{\mathrm{p}}\;\left(\mathrm{\mu A}\right)=2.97+190.31\; \mathrm{C }\;\left(\mathrm{\mu mol }{\mathrm{L}}^{-1}\right)& \left(r=0.9966\right)\left(\mathrm{for}\; 1\mathrm{st}\; \mathrm{range}\right)\end{array}$$

$$\begin{array}{cc}{I}_{\mathrm{p}}\;\left(\mathrm{\mu A}\right)=22.35+8.66\; \mathrm{C }\;\left(\mathrm{\mu \;mol\; }{\mathrm{L}}^{-1}\right)& \left(r=0.9979\right)\left(\mathrm{for} \;2\mathrm{nd} \;\mathrm{range}\right)\end{array}$$

LOD and LOQ are essential factors to consider while estimating the method. The results of the first linearity range were used to estimate the detection limit (3.3σ/S), which was determined to be 0.003 µmol L^−1^, and the quantitation limit (10σ/S), which was determined to be 0.009 µmol L^−1^, where the standard deviation of intercept is represented by *σ*, and the slope is represented by *S*. These results showed that the electrode could detect Trp in biological fluids even at very low concentrations.

By analyzing six samples (for intra-day study) and 18 samples at three successive days (for inter-day study) for a concentration of 7.0 μmol L^−1^, the proposed method’s intra-day and inter-day precision study is evaluated. The intra-day precision and inter-day precision % RSD values for the peak current of Trp were 2.09 and 3.33, respectively. The results demonstrate that the procedure can be regarded as accurate and reliable.

The robustness was also evaluated for the proposed method by estimation of the effect of little variations in the variables of the experiment on the recovery of the compound used (Trp). The robustness of the method indicates its ability to withstand these minor changes. Different parameters were studied (using 4.0 × 10^−5^ mol L^−1^ of Trp solution) such as medium pH (2.75 ± 0.25), accumulation potential (0.2 ± 0.01 V), and accumulation time (90 ± 2 s). Percentage recoveries in all experiments were ranged from 98.34 to 102.04 indicating that small changes in the method parameters have no impact on how well the proposed approach is performed in determining Trp and whether it can be used to analyze cancer patients’ serum in the future.

### Clinical applications

Based on recent studies, it was indicated that Trp metabolism is affected in breast cancer patients. This phenomenon is based on that catabolism of Trp increased through the kynurenine pathway leading to increased Kyn/Trp ratio [4, 9, 11–14, 68]. This information promotes the use of Trp as a biomarker for breast cancer females. Through our proposed method, we were able to estimate serum Trp concentrations in control and breast cancer females. Through these data, the Trp metabolic pathway may be a suitable target for monitoring the prognosis of breast cancer in females. Our proposed method was able to measure Trp in serum after dilution 20 times with B.R. buffer pH 2.75 without the need for any other sample treatment. Samples from healthy volunteers and breast cancer patients were analyzed to explore if there were significant differences in the level of Trp between the two groups.

**Fig. 7:**
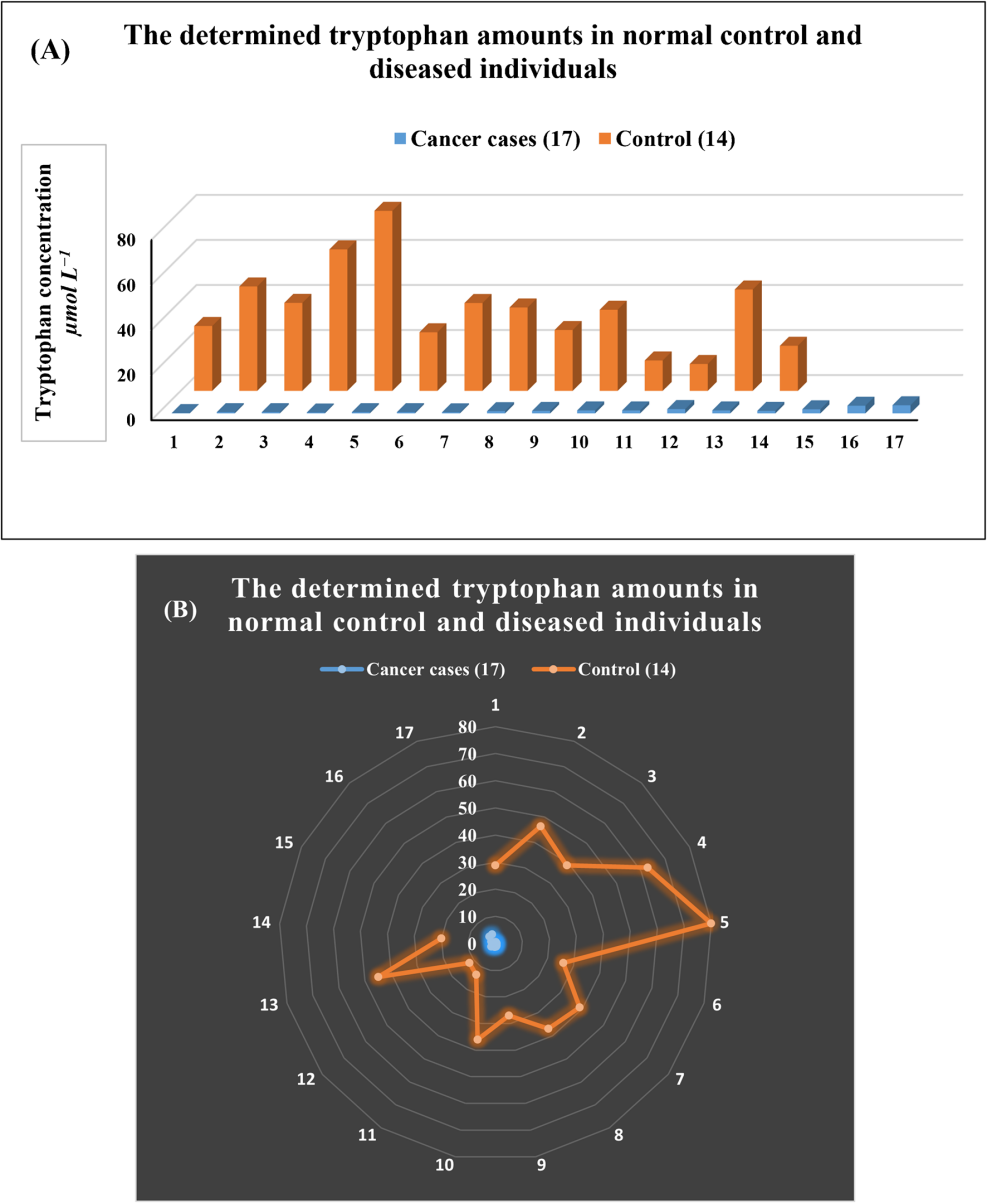
**A** Bar graphs for tryptophan serum concentrations in normal controls and diseased individuals. **B** Radar chart of tryptophan serum concentrations. The orange-colored section represents normal control while the blue-colored represents the diseased individuals

As is shown in Fig. 7A and B, compared with healthy volunteers, serum levels of total Trp in cancer patients decreased significantly. Table 2 shows that there was a significant difference between the results in healthy control subjects (*n* = 14) and cancer patients (*n* = 17) (*p* value = ˂ 0.05). The data were analyzed by ANOVA test and statistical comparisons between the group of diseased individuals (cancer patients) and the group of healthy individuals (control group) were performed using *F*-test and *t*-test. The *F*-test is used to compare the variances of the two groups while the *t*-test provides a useful tool for evaluating the differences in means between groups. It was found that the calculated *F*-value is greater than the critical *F*-value and it can be concluded that the variances are significantly different. It was found also that the calculated *t*-value is greater than the critical *t*-value and it can be concluded that the variances are significantly different. This confirms that the presence of cancerous cells is associated with increased variability in Trp serum level; therefore, it can be used as a biomarker in breast cancer cases. These results suggest the possibility of Trp as a biomarker and the modified Ov-Ox PPy/CDs/PGE as a biosensor for Trp in human blood serum.

**Table 2:** Statistical comparisons between control subjects and cancer patients

Statistical values	Control subjects (*n* = 14)	Cancer patients (*n* = 17)
*P*-value	9.8 × 10^−9^ ˂ 0.05	
*t*-test	*t*_calculated_ = 7.17, *t*_critical_ = 1.77	
F-test	*F*_calculated_ = 345.70, *F*_critical_ = 2.40	

### Selectivity and interference study

Selectivity study for the voltammetric determination of tryptophan in serum should aim to evaluate the ability of the method to accurately detect tryptophan in the presence of other potentially interfering substances that may be present in serum. We tested the ability of the proposed method to accurately and specifically evaluate Trp under the optimum conditions of the proposed method in the presence of potentially interfering substances such as other amino acids (glycine, alanine, phenylalanine, and tyrosine) or other small molecules such as ascorbic acid (AA) and uric acid (UA). This can be done by analyzing serum samples spiked with known concentrations of Trp and interfering substances at various ratios. It was found that 100-fold concentrations of glycine, alanine, phenylalanine, and 50-fold concentrations of (AA) and (UA) had no effect on Trp oxidation (signal change  ≤  ± 5.0%, high tolerance limit). However, tyrosine has an oxidation peak very close to Trp (0.70 V). The study found that even a fivefold concentration of tyrosine did not have a significant effect on the determination of Trp, which suggests that the method may still be suitable for the analysis of serum samples that contain low concentrations of tyrosine.

### Comparison of the proposed method with previously published methods

Compared to other analytical methods, such as chromatography, voltammetric techniques offer several advantages, including high sensitivity, selectivity, speed, cost-effectiveness, and minimal sample preparation. However, the choice of analytical method ultimately depends on the specific requirements of the application and the properties of the analyte being analyzed. The comparison of the newly proposed method and the previously published chromatographic (HPLC) methods is presented in Table 3. Overall, when compared with voltammetric methods, HPLC has several disadvantages for the analysis of biological samples, including extensive sample preparation (including extraction, filtration, and purification, to remove interfering substances from the sample), lack of selectivity, high cost, slow analysis time (depending on the complexity of the sample matrix and the separation method used), and limited sensitivity.

**Table 3:** Comparison of the proposed methods with some reported HPLC methods

Amino acids	Mobile phase	Detection	Linearity range	Limit of detection (LOD)	Matrix	Ref
Trp and Tyr	0.1 mol/l KH_2_PO_4_ and methanol (85:15, V/V)	Fluorescence detection	0.049–49.00 μmol/l	0.0049 μmol/l for Trp	Serum samples	[69]
Trp and its metabolites	Acetate buffer (pH 4.5) and acetonitrile	Uv detection at *λ*_max_ = 302 nm for Trp	3.97–400 μmol/L	0.134 μmol/L for Trp	Plasma samples	[25]
Trp and its metabolites	0.2 mol/L zinc acetate, 8.3 mmol/L acetate acid with 2.5% (v/v) acetonitrile	Fluorescence detection	0.245–196 μmol/L	0.001 μmol/L	Serum samples	[24]
Trp, Tyr, and Phe with other AAs	Mobile phase A: 1 M sodium acetate buffer pH 4.90 and ethanol Mobile phase D was a 1:50 dilution of 1 M sodium acetate buffer pH 5.85 Mobile phases B and C were acetonitrile and water respectively	UV detection at (250 nm)	5.88–188.05 μM	1.29 μM	Plasma samples	[70]
Trp and Tyr with their metabolites	Mobile phase A: ultrapure water plus 0.2% FA Mobile phase B: ACN plus 0.2% formic acid	Electrospray ionization triple quadrupole mass spectrometry	90–110,000 nM for Trp	40 nM	Human serum and cerebrospinal fluid	[71]
Trp, Tyr, and Phe with other AAs	Mobile phase A (named APDSTAG™ Wako Eluent) and mobile phase B (acetonitrile)	ESI mode mass spectrometry	6.25–250 μmol/L for Trp	0.44 μmol/Lfo Trp	Human plasma samples	[72]

When comparing the newly developed method with previous voltammetric studies, there are several factors to consider, including the sensitivity, selectivity, speed, cost-effectiveness, and ease of use of the new method compared with the previous methods. The newly developed method offers higher sensitivity than the other voltammetric studies, possibly due to the synergistic effect of carbon dots and polypyrrole film as indicated in Table 4. This may allow for more accurate detection of analytes at lower concentrations, which could be useful in applications if the high sensitivity is critical. The newly developed method also offers lower cost compared with previous voltammetric studies, possibly due to the use of simpler instrumentation or less expensive materials such as carbon dots that are easily prepared without high cost. This may make the method more accessible to researchers or clinicians who have limited resources.

**Table 4:** Comparison of the proposed methods with other voltammetric methods

Modified electrode	Matrix	Linearity range (μmol L^−1)^	Detection limit (μmol L^−1^)	Ref
(Au/Ag/Pd) NPs/EPGrO/GCE^**a**^	Human serum	1–600	0.03 ± 0.01	[73]
Cu_2_O–ERGO/GCE^**b**^	Human serum	0.02 to 20	0.01	[74]
ZnO/CPE^c^	Standard samples	10–40	0.57	[75]
NiO/CPE^**d**^	Standard samples	10 to 50	2.17	[76]
MWCNTs/IL/CPE^**e**^	Commercial amino acid injection and blood serum	1 to 1000	2.30	[77]
Pr-TiO_2_/f-CNT/SPCE^**f**^	Commercial amino acid dosage form	0.49 to 10.93 and 15.95 to 89.59	0.024	[78]
[C4mim]-[PF6]/Pt/CNTs/CPE^**g**^	Food and pharmaceutical samples	0.1–400.00	0.04	[28]
Ov-Ox PPy/CDs/PGE	Human serum	**0.01–0.09 and 0.50–9.00**	**0.003**	This work

The bold text indicate the results of the proposed work compared with the other work

^a^(Au/Ag/Pd) NPs capped with electropretreated graphene oxide (GO)–modified glassy carbon electrode (GCE); ^b^Cu_2_O–ERGO/GCE: Cu_2_O/electroreduced GO; ^c^zinc oxide nanoparticles modified carbon paste electrode; ^d^nickel oxide nanoparticles modified carbon paste electrode; ^e^multi-walled carbon nanotube–ionic liquid composite film modified carbon paste electrode; ^f^Pr-TiO_2_/f-CNT/SPCE: praseodymium-titanium oxide/acid functionalized carbon nanotubes/screen printed carbon electrode; ^**g**^[C4mim]-[PF6]: 1-butyl-3-methylimidazolium hexafluoro phosphate

## Conclusion

For the sensitive assessment of tryptophan in human serum in a short period of time (10 min), a new nanocomposite was developed by combining overoxidized polypyrrole polymer with nano-carbon dots on the surface of a pencil graphite electrode. The findings showed that the Ov-Ox PPy/CDs/PGE biosensor improved not only the electron transfer on the electrode’s surface but also the electrical conductivity and enhanced sensitivity of the electrochemical detection of tryptophan in samples of human serum.

The high porosity of the deposited layer and the ion exchange equilibrium that the ppy film brought are attributed to triggering this property. With all these benefits, the developed biosensor is ideal for the sensitive determination of Trp in serum from both healthy participants and female breast cancer patients. It can be considered as a simple, non-invasive, and non-sophisticated method for the detection of a certain type of disease, cancer as an example; we can one day get rid of the need for biopsies to detect cancer; however further studies are needed.

## Supplementary Information

Below is the link to the electronic supplementary material.

Supplementary file1 (DOCX 741 KB)

## References

[1] Palego L, Betti L, Rossi A, Giannaccini G. Tryptophan biochemistry: structural, nutritional, metabolic, and medical aspects in humans. J Amino Acids. 2016; 2016. 10.1155/2016/8952520 PMC4737446 26881063

[2] Bell C, Abrams J, Nutt D (2001). Tryptophan depletion and its implications for psychiatry. Br J Psychiatry.

[3] van Donkelaar EL, Kelly PA, Dawson N, Blokland A, Prickaerts J, Steinbusch HW (2010). Acute tryptophan depletion potentiates 3, 4-methylenedioxymethamphetamine-induced cerebrovascular hyperperfusion in adult male wistar rats. J Neurosci Res.

[4] Pietkiewicz D, Klupczynska-Gabryszak A, Plewa S, Misiura M, Horala A, Miltyk W (2021). Free amino acid alterations in patients with gynecological and breast cancer: a review. Pharmaceuticals (Basel)..

[5] Snedden W, Mellor CS, Martin JR (1983). Familial hypertryptophanemia, tryptophanuria and indoleketonuria. Clin Chim Acta.

[6] https://www.who.int/news-room/fact-sheets/detail/breast-cancer. Accessed 30 Jan 2023.

[7] Mitruka M, Gore CR, Kumar A, Sarode SC, Sharma NK (2020). Undetectable free aromatic amino acids in nails of breast carcinoma: biomarker discovery by a novel metabolite purification VTGE system. Front Oncol.

[8] Contorno S, Darienzo RE, Tannenbaum R (2021). Evaluation of aromatic amino acids as potential biomarkers in breast cancer by Raman spectroscopy analysis. Sci Rep.

[9] Prendergast GC (2011). Why tumours eat tryptophan. Nature.

[10] Peyraud F, Guegan JP, Bodet D, Cousin S, Bessede A, Italiano A. Targeting Tryptophan Catabolism in Cancer Immunotherapy Era: Challenges and Perspectives. Front Immunol. 2022;13. 10.3389/fimmu.2022.807271 PMC8841724 35173722

[11] Juhasz C, Nahleh Z, Zitron I, Chugani DC, Janabi MZ, Bandyopadhyay S (2012). Tryptophan metabolism in breast cancers: molecular imaging and immunohistochemistry studies. Nucl Med Biol.

[12] Lyon DE, Walter JM, Starkweather AR, Schubert CM, McCain NL (2011). Tryptophan degradation in women with breast cancer: a pilot study. BMC Res Notes.

[13] Puccetti P, Fallarino F, Italiano A, Soubeyran I, MacGrogan G, Debled M (2015). Accumulation of an endogenous tryptophan-derived metabolite in colorectal and breast cancers. PLoS ONE.

[14] Onesti CE, Boemer F, Josse C, Leduc S, Bours V, Jerusalem G (2019). Tryptophan catabolism increases in breast cancer patients compared to healthy controls without affecting the cancer outcome or response to chemotherapy. J Transl Med.

[15] Wu Y, Wang T, Zhang C, Xing X-H (2018). A rapid and specific colorimetric method for free tryptophan quantification. Talanta.

[16] Li H, Li F, Han C, Cui Z, Xie G, Zhang A (2010). Highly sensitive and selective tryptophan colorimetric sensor based on 4, 4-bipyridine-functionalized silver nanoparticles. Sensors Actuators B Chem.

[17] Othman AM, Li S, Leblanc RM (2013). Enhancing selectivity in spectrofluorimetric determination of tryptophan by using graphene oxide nanosheets. Anal Chim Acta.

[18] Zolfonoun E (2019). Spectrofluorometric determination of L-tryptophan after preconcentration using multi-walled carbon nanotubes. Anal Methods Environ Chem J.

[19] Abdolmohammad-Zadeh H, Oskooyi SMH (2015). Solid-phase extraction of l-tryptophan from food samples utilizing a layered double hydroxide nano-sorbent prior to its determination by spectrofluorometry. J Iran Chem Soc.

[20] Lesniak WG, Jyoti A, Mishra MK, Louissaint N, Romero R, Chugani DC (2013). Concurrent quantification of tryptophan and its major metabolites. Anal Biochem.

[21] Mazzucco E, Gosetti F, Bobba M, Marengo E, Robotti E, Gennaro MC (2010). High-performance liquid chromatography− ultraviolet detection method for the simultaneous determination of typical biogenic amines and precursor amino acids. Applications in Food Chemistry. J Agric Food Chem.

[22] Redruello B, Ladero V, Cuesta I, Álvarez-Buylla JR, Martín MC, Fernández M (2013). A fast, reliable, ultra high performance liquid chromatography method for the simultaneous determination of amino acids, biogenic amines and ammonium ions in cheese, using diethyl ethoxymethylenemalonate as a derivatising agent. Food Chem.

[23] Wang L, Xu R, Hu B, Li W, Sun Y, Tu Y (2010). Analysis of free amino acids in Chinese teas and flower of tea plant by high performance liquid chromatography combined with solid-phase extraction. Food Chem.

[24] Xiang Z-Y, Tang A-G, Ren Y-P, Zhou Q-X, Luo X-B (2010). Simultaneous determination of serum tryptophan metabolites in patients with systemic lupus erythematosus by high performance liquid chromatography with fluorescence detection. Clin Chem Lab Med.

[25] Zhen Q, Xu B, Ma L, Tian G, Tang X, Ding M (2011). Simultaneous determination of tryptophan, kynurenine and 5-hydroxytryptamine by HPLC: application in uremic patients undergoing hemodialysis. Clin Biochem..

[26] Corrêa de Carvalho R, Rocha dos Santos Mathias T, Duarte Pereira Netto A, Ferreira de Carvalho Marques F. Direct determination of amino acids in brewery worts produced by different processes by capillary zone electrophoresis. Electrophoresis 2018;39(13):1613–20. 10.1002/elps.201700327 29231974

[27] Deng P, Fei J, Feng Y (2011). Sensitive Voltammetric determination of tryptophan using an acetylene black paste electrode modified with a Schiff’s base derivative of chitosan. Analyst.

[28] Khaleghi F, Irai AE, Gupta VK, Agarwal S, Bijad M, Abbasghorbani M (2016). Highly sensitive nanostructure voltammetric sensor employing Pt/CNTs and 1-butyl-3-methylimidazolium hexafluoro phosphate for determination of tryptophan in food and pharmaceutical samples. J of Molecular Liquids.

[29] Kooshki M, Abdollahi H, Bozorgzadeh S, Haghighi B (2011). Second-order data obtained from differential pulse voltammetry: determination of tryptophan at a gold nanoparticles decorated multiwalled carbon nanotube modified glassy carbon electrode. Electrochim Acta.

[30] Mattioli IA, Baccarin M, Cervini P, Cavalheiro ÉT (2019). Electrochemical investigation of a graphite-polyurethane composite electrode modified with electrodeposited gold nanoparticles in the voltammetric determination of tryptophan. J of Electroanal Chem..

[31] Mohammadi SZ, Beitollahi H, Hassanzadeh M (2018). Voltammetric determination of tryptophan using a carbon paste electrode modified with magnesium core shell nanocomposite and ionic liquids. Anal Bioanal Chem Res.

[32] Saeidinejad F, Ghoreishi SM, Masoum S, Behpour M (2020). Application of chemometric methods for the voltammetric determination of tryptophan in the presence of unexpected interference in serum samples. Measurement.

[33] Shahrokhian S, Bayat M (2011). Pyrolytic graphite electrode modified with a thin film of a graphite/diamond nano-mixture for highly sensitive voltammetric determination of tryptophan and 5-hydroxytryptophan. Microchim Acta.

[34] Khan MZH, Liu X, Tang Y, Zhu J, Hu W, Liu X (2018). A glassy carbon electrode modified with a composite consisting of gold nanoparticle, reduced graphene oxide and poly (L-arginine) for simultaneous voltammetric determination of dopamine, serotonin and L-tryptophan. Microchimica Acta.

[35] Sun D, Li H, Li M, Li C, Dai H, Sun D, et al. Electrodeposition synthesis of a NiO/CNT/PEDOT composite for simultaneous detection of dopamine, serotonin, and tryptophan. 2018;259:433–42.

[36] Abdel-Aal FAM, Rageh AH, Said MI, Saleh GA (2018). epsilon-MnO_2_-modified graphite electrode as a novel electrochemical sensor for the ultrasensitive detection of the newly FDA approved Hepatitis C antiviral drug ledipasvir. Anal Chim Acta.

[37] Ali MF, Abdel-Aal FA (2019). In situ polymerization and FT-IR characterization of poly-glycine on pencil graphite electrode for sensitive determination of anti-emetic drug, granisetron in injections and human plasma. RSC Adv.

[38] Said MI, Rageh AH, Abdel-Aal FA (2018). Fabrication of novel electrochemical sensors based on modification with different polymorphs of MnO2 nanoparticles. Application to furosemide analysis in pharmaceutical and urine samples. RSC Adv.

[39] Liu J, Li R, Yang B (2020). Carbon dots: a new type of carbon-based nanomaterial with wide applications. ACS Cent Sci.

[40] Shankar SS, Shereema RM, Ramachandran V, Sruthi T, Kumar VS, Rakhi R (2019). Carbon quantum dot-modified carbon paste electrode-based sensor for selective and sensitive determination of adrenaline. ACS Omega.

[41] Zhuang X, Wang H, He T, Chen L (2016). Enhanced voltammetric determination of dopamine using a glassy carbon electrode modified with ionic liquid-functionalized graphene and carbon dots. Microchim Acta..

[42] Ermiş N, Tinkiliç N (2018). Preparation of molecularly imprinted polypyrrole modified gold electrode for determination of tyrosine in biological samples. Int J Electrochem Sci.

[43] Abdel-Hamid R, Newair EF (2015). Voltammetric determination of ferulic acid using polypyrrole-multiwalled carbon nanotubes modified electrode with sample application. Nanomaterials (Basel).

[44] Gong L, Li S, Yin Z, Li K, Gu J, Wu D (2021). Enantioselective recognition of tryptophan isomers with molecularly imprinted overoxidized polypyrrole/poly (p-aminobenzene sulfonic acid) modified electrode. Chirality.

[45] Dinu A, Apetrei C (2021). Development of a novel sensor based on polypyrrole doped with potassium hexacyanoferrate (II) for detection of L-tryptophan in pharmaceutics. Inventions.

[46] Ebarvia BS, Cabanilla S, Sevilla F (2005). Biomimetic properties and surface studies of a piezoelectric caffeine sensor based on electrosynthesized polypyrrole. Talanta.

[47] Sharma A, Das J (2019). Small molecules derived carbon dots: synthesis and applications in sensing, catalysis, imaging, and biomedicine. J Nanobiotechnology.

[48] Gao L, Wang Y, Lu M, Fa M, Yang D, Yao X (2017). Simple method for O-GlcNAc sensitive detection based on graphene quantum dots. RSC Adv.

[49] Hao J, Li L, Zhao W, Wu X, Xiao Y, Zhang H, et al. Carboxyl carbon quantum dots: a novel type of environmental-friendly scale inhibitor. 2018.

[50] Zheng M, Xie Z, Qu D, Li D, Du P, Jing X (2013). On-off-on fluorescent carbon dot nanosensor for recognition of chromium(VI) and ascorbic acid based on the inner filter effect. ACS Appl Mater Interfaces.

[51] Zhao C, Liang Y, Li W, Tian Y, Chen X, Yin D (2017). BiOBr/BiOCl/carbon quantum dot microspheres with superior visible light-driven photocatalysis. RSC Adv.

[52] Kudr J, Richtera L, Xhaxhiu K, Hynek D, Heger Z, Zitka O (2017). Carbon dots based FRET for the detection of DNA damage. Biosens Bioelectron.

[53] Gao MX, Liu CF, Wu ZL, Zeng QL, Yang XX, Wu WB (2013). A surfactant-assisted redox hydrothermal route to prepare highly photoluminescent carbon quantum dots with aggregation-induced emission enhancement properties. Chem Commun (Camb).

[54] Chunduri LAA, Kurdekar A, Patnaik S, Dev BV, Rattan TM, Kamisetti V (2016). Carbon quantum dots from coconut husk: evaluation for antioxidant and cytotoxic activity. Materials Focus.

[55] Orazem ME, Tribollet B. Electrochemical impedance spectroscopy. John Wiley & Sons; 2017.

[56] Wang J, Lu J. Electrochemical impedance spectroscopy: fundamentals and applications. John Wiley & Sons; 2019.

[57] Abdelhamid HN, Kailasa SK, Hussain CM (2023). Chapter 7 - Carbon dots for electrochemical analytical methods. Carbon dots in analytical chemistry.

[58] Ansari R (2006). Polypyrrole conducting electroactive polymers: synthesis and stability studies. E-J Chem.

[59] Li CM, Sun CQ, Chen W, Pan L (2005). Electrochemical thin film deposition of polypyrrole on different substrates. Surf Coat Technol.

[60] Pang AL, Arsad A, Ahmadipour M (2021). Synthesis and factor affecting on the conductivity of polypyrrole: a short review. Polym Adv Technol.

[61] Sakthivel R, Mutharani B, Chen S-M, Kubendhiran S, Chen T-W, Al-Hemaid FMA (2018). A simple and rapid electrochemical determination of L-tryptophan based on functionalized carbon black/poly-L-histidine nanocomposite. J Electrochem Soc.

[62] Oikawa Y, Yonemitsu O (1977). Selective oxidation of the side chain at C-3 of indoles. J Org Chem.

[63] Laviron E (1979). General expression of the linear potential sweep voltammogram in the case of diffusionless electrochemical systems. J Electroanal Chem Interf Electrochem.

[64] Wang Y, Ouyang X, Ding Y, Liu B, Xu D, Liao L (2016). An electrochemical sensor for determination of tryptophan in the presence of DA based on poly(l-methionine)/graphene modified electrode. RSC Adv.

[65] Zhou S, Deng Z, Wu Z, Xie M, Tian Y, Wu Y, et al. Ta_2_O_5_/rGO Nanocomposite modified electrodes for detection of tryptophan through electrochemical route. Nanomaterials. 2019;9(6). 10.3390/nano9060811 PMC6631568 31142057

[66] Lima D, Andrade Pessôa C, Wohnrath K, Humberto Marcolino-Junior L, Fernando Bergamini M. A feasible and efficient voltammetric sensor based on electropolymerized L-arginine for the detection of L-tryptophan in dietary supplements. Microchem J. 2022;181.

[67] Tasić ŽZ, Petrović Mihajlović MB, Radovanović MB, T. Simonović AT, V. Medić DV, Antonijević MM. Electrochemical determination of L-tryptophan in food samples on graphite electrode prepared from waste batteries. Sci Rep. 2022;12:5469. 10.1038/s41598-022-09472-7 PMC8971531 35361843

[68] Pietkiewicz D, Klupczynska-Gabryszak A, Plewa S, Misiura M, Horala A, Miltyk W, et al. Free amino acid alterations in patients with gynecological and breast cancer: a review. Pharmaceuticals (Basel). 2021;14(8). 10.3390/ph14080731 PMC8400482 34451829

[69] Sa M, Ying L, Tang A-G, Xiao L-D, Ren Y-P (2012). Simultaneous determination of tyrosine, tryptophan and 5-hydroxytryptamine in serum of MDD patients by high performance liquid chromatography with fluorescence detection. Clin Chim Acta.

[70] Wang H, McNeil YR, Yeo TW, Anstey NM (2013). Simultaneous determination of multiple amino acids in plasma in critical illness by high performance liquid chromatography with ultraviolet and fluorescence detection. J Chromatogr B Analyt Technol Biomed Life Sci.

[71] Galla Z, Rajda C, Rácz G, Grecsó N, Baráth Á, Vécsei L, et al. Simultaneous determination of 30 neurologically and metabolically important molecules: a sensitive and selective way to measure tyrosine and tryptophan pathway metabolites and other biomarkers in human serum and cerebrospinal fluid. J Chromatogr A. 2021;1635:461775. 10.1016/j.chroma.2020.461775 33302138

[72] Yoshida H, Kondo K, Yamamoto H, Kageyama N, Ozawa S-i, Shimbo K, et al. Validation of an analytical method for human plasma free amino acids by high-performance liquid chromatography ionization mass spectrometry using automated precolumn derivatization. J Chromatogr B. 2015;998:88–96. 10.1016/j.jchromb.2015.05.029 26186723

[73] Abdelwahab AA, Elseman A, Alotaibi N, Nassar AM. Simultaneous voltammetric determination of ascorbic acid, dopamine, acetaminophen and tryptophan based on hybrid trimetallic nanoparticles-capped electropretreated graphene. Microchemical J. 2020;156:104927.

[74] He Q, Tian Y, Wu Y, Liu J, Li G, Deng P (2019). Electrochemical sensor for rapid and sensitive detection of tryptophan by a Cu2O nanoparticles-coated reduced graphene oxide nanocomposite. Biomolecules.

[75] Vishwanath MS, Swamy BK, Vishnumurthy KA (2023). Zinc oxide modified carbon paste electrode sensor for the voltammetric detection of L-tryptophan in presence of uric acid and ascorbic acid. Inorg Chem Commun.

[76] Swamy B, Murthy V (2022). Nickel oxide modified carbon paste electrode for the cyclic voltammetric detection of L-tryptophan and uric acid. Anal Bioanal Electrochem.

[77] Rezaee E, Honarasa F (2018). Determination of tryptophan by using of activated multi-walled carbon nanotube ionic liquid electrode. Russ J Electrochem.

[78] Gopi PK, Subburaj S, Chen S-M, Chia-Jung W, Ravikumar CH (2021). Pr-TiO2 decorated functionalized-carbon nano tubes for highly selective detection of tryptophan in pharmaceutical samples for neurotransmitter treatment. J Electrochem Soc..

