# Supplementary material for: Polypyrrole/carbon dot nanocomposite as an electrochemical biosensor for liquid biopsy analysis of tryptophan in the human serum of normal and breast cancer women
Source: Anal Bioanal Chem. 2023 Jul 4;415(20):4985–5001. doi: 10.1007/s00216-023-04784-7 (PMC10386971; doi:10.1007/s00216-023-04784-7)
Supplement: Supplementary file 1 — Supplementary file1 (DOCX 741 KB) [file 216_2023_4784_MOESM1_ESM.docx]

**Polypyrrole/carbon dot nanocomposite as an electrochemical biosensor for liquid biopsy analysis of
tryptophan in the human serum of normal and breast cancer women**

**Fatma A. M. Abdel-aal *^a^, Rania M. Kamel ^a^, Asmaa A. Abdeltawab ^b^,** **Fardous A. Mohamed ^a^, Abdel-Maaboud I. Mohamed ^a^**

**^a^ Pharmaceutical Analytical Chemistry Department, Faculty of Pharmacy, Assiut University, 71526 Assiut, Egypt.**

**^b^ Clinical Oncology and Nuclear medicine Department, Faculty of Medicine, Assuit University, 71526 Assiut, Egypt**













**Fig. 1S: Cyclic voltammograms of 1.0 mmol L^-1^ K_3_[Fe(CN)_6_] in 0.5 mol L^-1^ KCl obtained at different scan rates (0.01 – 0.90 Vs^-1^) using (A) Bare PGE, (B) CDs/PGE, (C) PPy/PGE, and (D) Ov-PPy/CDs/PGE,** **accumulation time, 60 s.**





**Fig. 2S: Effects of (A) pyrrole conc., (B) polymerization potential, and (C) polymerization cycles on the peak current of 4.0 ×10^-5^ mol L^-1^ Trp solution.**





**Fig. 3S: Effect of (A) accumulation potential (from -0.5 to 0.4 V) and (B) accumulation time from 15 to 150 sec. on the oxidation peak of 4.0 × 10^-5^ mol L^-1^ Trp solution.**
